# Serum Concentrations and Dietary Intake of Vitamin B_12_ in Children and Adolescents on Metformin: A Case–Control Study

**DOI:** 10.3390/ijms24044205

**Published:** 2023-02-20

**Authors:** Kyriaki Tsiroukidou, Eleni G. Paschalidou, Maria G. Grammatikopoulou, John Androulakis, Anastasios Vamvakis, Kalliopi K. Gkouskou, Christos Tzimos, Theodoros N. Sergentanis, Tonia Vassilakou, Emmanuel Roilides, Dimitrios P. Bogdanos, Dimitrios G. Goulis

**Affiliations:** 1 3rd Department of Pediatrics, Hippokration General Hospital of Thessaloniki, Aristotle University of Thessaloniki, GR-54124 Thessaloniki, Greece; 2 Department of Rheumatology and Clinical Immunology, University General Hospital of Larissa, Faculty of Medicine, School of Health Sciences, University of Thessaly, Biopolis, GR-41110 Larissa, Greece; 3 IsoPlus, Scientific Department, 236 Syggrou Avenue, GR-17672 Athens, Greece; 4 Department of Nutrition and Dietetics, Sciences School of Health Science, Hellenic Mediterranean University, GR-71410 Heraklion, Greece; 5 Laboratory of Biology, School of Medicine, National and Kapodistrian University of Athens, GR-11527 Athens, Greece; 6 Northern Greece Statistics Directorate, Hellenic Statistical Authority, 218 Delfon Str., GR-54646 Thessaloniki, Greece; 7 Department of Public Health Policy, School of Public Health, University of West Attica, GR-11521 Athens, Greece; 8 Unit of Reproductive Endocrinology, 1st Department of Obstetrics and Gynecology, Faculty of Health Sciences, Medical School, Aristotle University of Thessaloniki, GR-54124 Thessaloniki, Greece

**Keywords:** one-carbon metabolism, childhood obesity, sulfonylurea, polycystic ovary syndrome, insulin resistance, Glucophage, pharmacotherapy, prediabetes, menstrual disorders, methyl-donor nutrient

## Abstract

The International Society of Pediatric and Adolescent Diabetes (ISPAD) recommends metformin (MET) use for metabolic disturbances and hyperglycemia, either in combination with insulin therapy or alone. A caveat of MET therapy has been suggested to be biochemical vitamin B_12_ deficiency, as seen mainly in studies conducted in adults. In the present case–control study, children and adolescents of different weight status tiers on MET therapy for a median of 17 months formed the cases group (*n* = 23) and were compared with their peers not taking MET (*n* = 46). Anthropometry, dietary intake, and blood assays were recorded for both groups. MET group members were older, heavier, and taller compared with the controls, although BMI z-scores did not differ. In parallel, blood phosphorus and alkaline phosphatase (ALP) concentrations were lower in the MET group, whereas MCV, Δ_4_-androstenedione, and DHEA-S were higher. No differences were observed in the HOMA-IR, SHBG, hemoglobin, HbA1c, vitamin B_12_, or serum 25(OH)D_3_ concentrations between groups. Among those on MET, 17.4% exhibited vitamin B_12_ deficiency, whereas none of the controls had low vitamin B_12_ concentrations. Participants on MET therapy consumed less energy concerning their requirements, less vitamin B_12_, more carbohydrates (as a percentage of the energy intake), and fewer fats (including saturated and trans fats) compared with their peers not on MET. None of the children received oral nutrient supplements with vitamin B_12_. The results suggest that, in children and adolescents on MET therapy, the dietary intake of vitamin B_12_ is suboptimal, with the median coverage reaching 54% of the age- and sex-specific recommended daily allowance. This low dietary intake, paired with MET, may act synergistically in reducing the circulating vitamin B_12_ concentrations. Thus, caution is required when prescribing MET in children and adolescents, and replacement is warranted.

## 1. Introduction

Metformin (1,1-dimethylbiguanide hydrochloride, MET) is considered the first-line oral blood glucose-lowering agent for the management of type 2 diabetes mellitus (T2DM) [1]. Although its use stems back to the Medieval European herbal medicine remedies of the plant *Galega officinalis* [2], the glucose-lowering properties of the plant were only discovered in 1918, attributed to its high guanidine content [3]. This propelled research on the use of guanidine derivatives, including MET, for the treatment of hyperglycemia. However, it was only in 1994 that the US Food and Drug Administration (FDA) first approved MET [1] due to the wide availability of insulin and the toxicity associated with guanidine use.

According to the American Diabetes Association (ADA), MET has been the drug of choice for the treatment of T2DM since the 1950s, carrying the “strongest evidence base” [4]. As for younger patients with T2DM, the International Society of Pediatric and Adolescent Diabetes (ISPAD) [5] recommends initial pharmacologic treatment with MET and insulin, either alone or in combination, depending on the degree of metabolic disturbances, as well as hyperglycemic and ketosis incidence. Furthermore, MET use can normalize the ovulatory abnormalities observed in girls with polycystic ovary syndrome (PCOS) [5]. More recently, research highlighted various MET-related extra-glycemic clinical benefits, including endothelial-protective [6], antineoplastic [7], and antiaging/anti-inflammatory effects [8]. Nonetheless, according to the ADA, long-term MET administration may be associated with biochemical vitamin B_12_ deficiency [4], and this has also been mentioned in the ISPAD guidelines [5].

MET consists of a complex I mitochondrial inhibitor [nicotinamide adenine dinucleotide (NADH): ubiquinone oxidoreductase], transported inside the cell to influence cellular respiration [9]. Complex I oxidizes the NADH that is synthesized from the one-carbon metabolism, fatty acid β-oxidation, glycolysis, and the tricarboxylic acid (TCA) cycle for the production of adenosine triphosphate (ATP) through the transport chain of electrons [9,10,11]. Thus, biguanides induce a partial inhibition of the ubiquinone reduction [10], which increases the accumulation of NADH and the synthesis of reactive oxygen species (ROS), limiting the production of ATP, while increasing the adenosine monophosphate (AMP):ATP ratio [9]. In turn, the increasing concentrations of AMP:ATP stimulate the AMP-activated protein kinase (AMPK), inhibiting gluconeogenesis, while maintaining euglycemia [10].

MET has been shown to influence the status of several micronutrients, including vitamin B_12_ and folate, both of which are important cofactors of the one-carbon metabolism [12,13]. In particular, MET use has been suggested to impair one-carbon metabolism similarly to anti-folate-class chemotherapy drugs administered for cancer [12,14]. Administration of MET reduces the intestinal absorption of vitamin B_12_, and the observed depression in vitamin B_12_ concentrations disturbs the methylation cycle, increasing total homocysteine (tHcy) concentrations [9]. In parallel, MET’s anti-folate activity also impairs the folate cycle [9]. Observational studies suggest that the administration of MET is associated with a small reduction in serum vitamin B_12_ concentrations, although contradictory findings have also been reported in the literature [13,15,16]. Furthermore, most data stem from studies conducted on adults, with only a few using populations of younger patients [17,18]. Even more worrying is the fact that children and adolescents with overweight and obesity appear to be at greater risk for developing vitamin B_12_ deficiency and exhibiting suboptimal vitamin B_12_ status, irrespective of MET use [19]. In this manner, children with obesity on MET treatment may face a dual risk for exhibiting lower vitamin B_12_ concentrations, as a result of the excessive body weight (BW) accumulation and the use of MET.

With this in mind, the present case–control study was designed to evaluate vitamin B_12_ status (dietary intake and serum levels) in adolescents on MET treatment, compared with their peers who were not receiving MET.

## 2. Results

### 2.1. Vitamin B_12_ Status, Hormonal Assays, and Vitamin D

Table 1 details the results of the blood assays in each study group. No differences were noted in the median vitamin B_12_, 25-hydroxyvitamin D_3_ [25(OH)D_3_], fasting glucose, and insulin concentrations, glycosylated hemoglobin (HbA1c), or the homeostatic model assessment of insulin resistance (HOMA-IR) between groups. On the other hand, more participants on MET demonstrated biochemical vitamin B_12_ deficiency compared with controls. Phosphorus and alkaline phosphatase (ALP) concentrations were lower in the MET group, whereas the Δ_4_-androstenedione, mean corpuscular volume (MCV) and dehydroepiandrosterone sulfate (DHEA-S) concentrations were higher.

### 2.2. Dietary Intake Results

Table 2 presents the recorded daily dietary intake of participants. The consumption of energy and fats, including total monounsaturated fatty acids (MUFA), saturated fatty acids (SFA), and trans fats, was greater among participants in the MET group compared with controls. Concerning vitamin intake, differences were only noted in the intake of vitamins B_6_ and B_12_, with a greater recorded intake among MET-receiving children, as well as concerning vitamin E, consumed in greater amounts than the controls. Iron, Magnesium, Zinc, and Sodium were also consumed in greater amounts by the controls.

The energy intake was suboptimal among controls, reaching 60% of their energy expenditure requirements (EER). Participants in either group failed to meet the recommendations for the intake of n-3 fatty acids, with those on MET therapy covering 39% (IQR 25.0–59.0) of their requirements and controls meeting 66.5% (IQR 49.0–90.5) of their needs. The dietary intake of vitamins B_9_, A, D, and E, Iron, Magnesium, Phosphorus, and Zinc was suboptimal among all participants, irrespective of group allocation. None of the participants in the MET-receiving arm reported taking oral nutrient supplements (ONS), whereas three participants (7%) belonging to the control group consumed multivitamin (MV) supplements.

## 3. Discussion

The present case–control study failed to show differences in the vitamin B_12_ concentrations between children and adolescents on MET therapy compared with controls. However, more participants on MET demonstrated biochemical vitamin B_12_ deficiency despite the greater reported dietary vitamin B_12_ intake.

The recommendations for the parallel administration of vitamin B_12_ in patients receiving MET were initiated from the results of several early case–control studies and clinical trials administering MET. One of these included the Diabetes Prevention Program (DPP) randomized controlled trial (RCT), where people with prediabetes used either MET or placebo [21]. A post hoc analysis of the data indicated a 13% increased risk of vitamin B_12_ deficiency/year of MET administration after 13 years of follow-up [21]. In another placebo-controlled RCT, people with T2DM on insulin were randomized to MET as an add-on therapy or placebo for a total of 4 years [22]. The results revealed a 19% decrease in B_12_ concentrations and a number needed to harm (NNH) of 13.8, over 4.3 years of follow-up [22]. Moreover, the reduction in B_12_ concentrations was not transitory, but persisted and progressed over time [22]. With this in mind, the UK Medicines and Healthcare products Regulatory Agency (MHRA) published a new guidance identifying low B_12_ concentrations as a distinct and common side-effect of MET therapy, especially among patients on high-doses or long-term treatment, estimated to affect up to one in 10 people [23]. In parallel, it recommends frequently checking B_12_ concentrations in patients with possible symptoms of deficiency and closely monitoring those at risk of deficiency [23].

The present study revealed a greater prevalence of biochemical vitamin B_12_ deficiency among children and adolescents on MET therapy compared to controls. Table 3 details the studies evaluating vitamin B_12_ status in children/adolescents on MET treatment. The results appear controversial. Some studies reported a lack of change in the vitamin B_12_ concentrations of minors on MET treatment [24,25,26], whereas others suggested that greater MET doses and prolonged treatment duration were associated with lower vitamin B_12_ concentrations [17,18,27,28,29], as suggested by the MHRA [23]. However, herein, the prevalence of vitamin B_12_ deficiency greatly exceeded the rate proposed by the MHRA [23], with 18% of the sample treated with MET exhibiting total vitamin B_12_ concentrations indicative of biochemical deficiency.

The lack of consensus regarding the exact definition of vitamin B_12_ deficiency is apparent in the scientific bubble [32,33]. The ongoing debate involves both the specific thresholds and the ideal biomarker (or combination of) to assess vitamin B_12_ status accurately [32,33,34]. Suggested circulating vitamin B_12_ biomarkers include total vitamin B_12_ or holo-transcobalamin (HoloTC), whereas metabolic biomarkers of vitamin B_12_ status involve the methylmalonic acid (MMA) or tHcy concentrations [35]. Several researchers [32,34,36] have underlined the limited diagnostic value of serum vitamin B_12_ concentrations, due to its low sensitivity and specificity in identifying true tissue vitamin B_12_ deficiency. In parallel, the assessment of total serum vitamin B_12_ concentrations includes the circulating levels of the vitamin, 80% of which is bound to haptocorrin, limiting its cellular uptake bioavailability [32].

Research conducted on adults on MET therapy has also revealed important associations with specific methylene tetrahydrofolate reductase (*MTHFR*) polymorphisms. In particular, the C677T *MTHFR* defect has the potential to increase Hcy concentrations due to lower levels of methylcobalamin and methylfolate. In this manner, MET users harboring the rs180133 677C > T have been shown to attain suboptimal vitamin B_12_ status, as well as greater Hcy and MMA levels [37,38]. For those carrying the C677T *MTHFR* variant, concomitant ONS with vitamin B_12_ and methylfolate is recommended to correct for the low circulating levels.

MET has also been suggested to inhibit Ca-dependent absorption of the vitamin’s B_12_ intrinsic factor complex, at the site of the terminal ileum [39]. Since MET use reduces B_12_ absorption through a Ca-dependent ileal membrane antagonism, Bauman [40] suggested that using Ca ONS can reverse the MET-induced serum vitamin B_12_ and HoloTC depression. Nonetheless, in the present study, none of the participants in the NET-receiving arm were taking Ca ONS, while, on the other hand, Ca dietary intake was suboptimal for all participants.

Recent research revealed that children and adolescents receiving high levels of dietary methyl-donor nutrients (including vitamin B_12_) have fewer chances of being metabolically unhealthy obese [41]. In parallel, vitamin B_12_ levels are inversely related to the metabolic risk score of both children and their parents, through arterial blood pressure, high-density lipoprotein (HDLc) cholesterol, and triglyceride levels [42]. Other researchers have shown a negative relationship between obesity and vitamin B_12_ concentrations [19] and an inverse association between IR and vitamin B_12_ concentrations [43] in children and adolescents, irrespective of MET use. According to Infante [32], many conditions can increase the risk of vitamin B_12_ deficiency, including inadequate dietary intake, impaired intrinsic factor secretion, malnutrition, vegetarianism, bacterial overgrowth syndromes, intestinal parasitic infestations, or disorders of the exocrine pancreas. All these factors should be evaluated prior to the initiation of MET therapy [32].

In the present study, participants in both groups reported a suboptimal dietary intake regarding several nutrients, with patients on MET therapy reporting the adoption of a more atherogenic diet. Furthermore, children and adolescents on MET treatment exhibited a greater consumption of dietary vitamin B_12_ through food, without any intake of ONS. Thus, the lack of difference in serum vitamin B_12_ concentrations between participants on MET therapy versus controls might well have resulted from increased dietary intake among the first. In parallel, it is also possible that the recorded greater vitamin B_12_ consumption might have corrected possible lower levels, resulting from prolonged or high-dose MET therapy. Nonetheless, despite the greater intake, more children and adolescents on MET therapy demonstrated inadequate circulating vitamin B_12_ levels compared to controls.

Concerning vitamin D concentrations, the present results confirmed previous studies on the fact that vitamin D status does not consist of a clinical concern among MET-treated patients [44,45].

An important limitation of the present study involves the relatively small sample size, as a larger population would probably have allowed reaching a significant difference in the concentration of vitamin B_12_ between the two groups analyzed. The use of total serum vitamin B_12_ concentrations as the only biomarker for the assessment of vitamin B_12_ status is another limitation. Nonetheless, most of the studies available in the literature (Table 3) also relied on serum vitamin B_12_ levels only [17,24,25,26,27,29,31]. Furthermore, the diet record of participants is an important addition to the assessment of vitamin B_12_ status, as most studies (Table 3) only relied on hematological parameters, ignoring the importance of dietary intake. Last, but not least, *MTHFR* polymorphisms were not assessed in the present population due to lack of consent by the parents, although they may well have impacted the observed associations.

Notably, suboptimal vitamin B_12_ status is not the only adverse event associated with MET use. Gastrointestinal symptoms, bloating, flatulence, nausea, and diarrhea have also been reported and appear to be dose-dependent [46].

## 4. Materials and Methods

### 4.1. Study Population

During the first half of the year 2022, pediatric and adolescent outpatients were recruited randomly in a convenient manner from the Pediatric Endocrinology Unit of the Third Department of Pediatrics, situated at Hippokration General Hospital in Thessaloniki, Greece.

Those on MET treatment formed the case study arm, while those who were not on MET served as the controls of the study. Controls were also selected randomly from the children and adolescents visiting the Pediatric Endocrinology Unit, due to premature adrenarche, thelarche, precocious puberty, idiopathic short stature, microphallus, gynecomasty, overweight/obesity, hypothyroidism, or evaluation of thyroid dysfunction. Outpatients on MET (cases) were diagnosed with overweight/obesity, menstrual disorders/PCOS, and/or prediabetes/insulin resistance (IR). The ratio of cases versus controls was set at 1:2. Participant characteristics in each study group are presented in Table 4.

### 4.2. Ethical Permission

Permission for the study was granted by the Scientific Committee of the Hippokration General Hospital (4694/31-01-2023). In parallel, the parents/guardians of the participating children provided consent prior to their child’s participation. The nature and purpose of the study were explained to all participants and their families by an experienced pediatric endocrinologist (K.T. and a dietitian E.G.P.)

All data were handled with emphasis on anonymity and data protection, according to the Declaration of Helsinki and its latter amendments.

### 4.3. Anthropometric Measurements

The BW and stature of participants were measured to the nearest g and cm, respectively, using a Seca 700 mechanical scale (Seca, Hamburg, Germany) and a Harpenden wall-mounted stadiometer (Holtain, Crymych, UK). All anthropometric measurements were performed in the morning by an experienced dietitian (E.G.P.).

Body mass index (BMI) was calculated for each participant as the ratio of BW (kg) to the square of height (m^2^). BMI z-scores (BMIz) were calculated using the World Health Organization (WHO) Anthro software v.3.2 (WHO, Geneva, Switzerland) [48] for the assessment of growth and development of children and adolescents, based on the WHO child growth standards and growth curves [47,49].

### 4.4. Dietary Intake

For each child/adolescent, a detailed previous 24 h diet recall was collected with the facilitation of food photos with realistic sizes, and the intake was analyzed using the Cronometer software (Cronometer Software Inc., Vancouver, BC, Canada) [50].

In parallel, the intake of ONS was recorded for all participants, and each nutrient’s daily intake was added to the respective recorded dietary intake.

### 4.5. Blood Samples and Assays

For biochemical and hormone profile analysis, fresh, whole-blood samples (20 mL) were collected from each participant in the morning hours, after overnight fasting. Plasma was isolated using ethylenediaminetetraacetic acid (EDTA). For serum isolation, whole blood was previously allowed to clot at room temperature for 20 min. Whole-blood samples were centrifuged at 3000 rpm for a total of 10 min at a temperature of 4 °C.

Immunoassay was performed via chemiluminescent detection for vitamin B_12_, total 25(OH)D_3_, insulin, sex hormone-binding globulin (SHBG), and dehydroepiandrosterone sulfate (DHEA-S). Insulin levels were assessed using a Human Insulin ELISA kit (ALPCO Diagnostics) with inter- and intra-assay precision below 15%.

The enzymatic method was used to assess blood glucose levels using an Abbot ALinity I analyzer. Ca, P, and ALP concentrations were estimated using Abbot ALinity C Analyzer (Abbott, Abbott Park, Chicago, IL, USA). Androstenedione was analyzed using an Immulite Siemens analyzer (Siemens Healthcare GmbH, Erlangen, Germany).

HbA1c (%) was assessed with a Siemens DCA Vantage analyzer (Siemens Healthcare GmbH, Erlangen, Germany).

#### 4.5.1. Insulin Resistance (IR)

Whole-body insulin sensitivity was evaluated with the homeostatic model assessment of insulin resistance (HOMA-IR) index in the fasting state [20], calculated as the fasting serum insulin concentrations (μU/mL) × plasma fasting glucose levels (mmol/L)/22.5.

#### 4.5.2. Vitamin B12 Deficiency

When serum vitamin B_12_ levels were below 140 pg/mL, biochemical vitamin B_12_ deficiency was diagnosed [28,30].

### 4.6. Statistical Analyses

Normality in the distribution of variables was assessed using the Kolmogorov–Smirnov and Shapiro–Wilk tests. None of the variables appeared to follow a normal distribution. Qualitative variables were expressed as medians with their interquartile ranges (first and third IQR), and qualitative variables were expressed as counts (*n*) with their respective percentages (%).

Between-group comparisons were conducted using the Mann–Whitney U or the chi-squared test. Fischer’s exact test was applied to compare frequencies between the two groups.

For the analyses, two statistical software packages were used (IBM Corp. Released 2021. IBM Statistical Package for Social Sciences (SPSS) for Windows, Version 28.0, Armonk, NY, USA: IBM Corp; the Jamovi project version 1.2.27.0) [51]. The significance level was set at 5% (α = 0.05) for all analyses.

## 5. Conclusions

The rises in the prevalence of childhood and adolescent overweight and obesity [52,53,54] and its associated comorbidities have increased the number of youngsters receiving MET therapy [55]. Overall, MET consists of a low-cost option with modest clinical benefits for BW loss and minimal side-effects [56,57]. Thus, when lifestyle treatment has been pursued as a first-line therapy and deemed suboptimal in achieving adequate weight loss, a reasonable continuum would be using MET, as an adjunctive therapy [56]. Nonetheless, MET treatment appears to affect vitamin B_12_ status in children and adolescents, irrespective of dietary intake. According to the recent clinical practice guidelines published by the American Academy of Pediatrics [58], when prescribing such medications, healthcare professionals must adequately inform patients and their parents about the risks and benefits of therapies and must have updated knowledge of the patient selection criteria, medication efficacy, possible adverse events, and the recommendations regarding follow-up monitoring. For this, frequent assessment of vitamin B_12_ concentrations and recording of symptoms and signs associated with vitamin B_12_ deficiency are warranted when prescribing MET to children and adolescents. In parallel, currently, there is no evidence supporting weight loss or diabetes medication use as a monotherapy [58]. In this manner, lifestyle treatment must be prescribed as an adjunct to MET therapy.

## Figures and Tables

**Table 1:** Results of the blood assays of participating children in each arm *.

	MET (*n* = 23)	Controls (*n* = 46)	*p*-Value
Vitamin Β_12_ (pg/mL)	284 (197–421)	363 (235–522)	0.135
Biochemical vitamin B_12_ deficiency (*n,* %) ^†^	4 (17%)	0 (0%)	0.016 ^§^
Total serum 25(OH)D_3_ (ng/mL)	26.0 (22.7–30.4)	25.5 (17.7–28.3)	0.330
Fasting glucose (mg/dL)	88 (81–95)	86 (82–93)	0.994
Fasting insulin (μIU/mL)	10.9 (7.7–18)	9.4 (8.1–13.8)	0.485
HOMA-IR	2.4 (1.8–4)	2 (1.5–2.8)	0.357
Hb (g/dL)	13.3 (12.6–14.5)	12.9 (12.3–13.7)	0.116
HbA1c (%)	5.1 (5–5.3)	5.2 (4.9–5.4)	0.676
MCV (fl)	86.1 (83.5–89.3)	81.8 (79.2–85.8)	0.001
Ca^2+^ (mg/dL)	10.0 (9.6–10.2)	9.8 (9.5–10)	0.197
P (mg/dL)	3.9 (3.6–4.3)	4.7 (4.0–5.1)	0.001
ALP (IU/L)	91 (79–128)	230 (132–294)	0.000
SHBG (nmol/L)	22.9 (15.2–37.6)	30.2 (16.5–38.3)	0.626
Δ_4_-Androstenedione (ng/mL)	2.1 (1.4–2.9)	0.7 (0.4–1.2)	<0.001
DHEA-S (μg/dL)	264 (225–334)	171 (88–224)	<0.001

25(OH)D_3_—25-hydroxyvitamin D_3_; ALP—alkaline phosphatase; Ca^2+^—Calcium; DHEA-S—dehydroepiandrosterone sulfate; Hb—hemoglobin; HbA1c—glycosylated hemoglobin; HOMA-IR—homeostatic model assessment of insulin resistance [20]; IQR—interquartile range; MCV—mean corpuscular volume; MET—metformin; P—phosphorus; SHBG—sex hormone-binding globulin. * Data are presented as medians with their respective first and third IQRs, or as counts (*n*) with their respective percentages (%); ^†^ vitamin B_12_ concentrations <140 pg/mL; ^§^ Fisher’s exact test.

**Table 2:** Daily dietary intake of participating children in each arm *.

Daily Nutrient Intake	MET (*n* = 23)	Controls (*n* = 46)	*p*-Value
Energy intake (EI) (kcal/day)	1522 (1183–1814)	1162 (823–1452)	0.004
Estimated energy expenditure (EER) (kcal/day)	1753 (1471–1969)	2008 (1699–2171)	0.007
EI (% of EER)	99.4 (58.5–119.0)	59.8 (40.3–74.2)	0.001
Carbohydrates (% of the EI)	45 (36–50)	50 (40–53)	NS
Proteins (% of the EI)	16 (13–18)	15 (13–21)	NS
Fats (% of the EI)	40 (35–45)	33 (29–43)	0.032
MUFA (g)	20 (16–32)	14 (10–19)	0.004
PUFA (g)	6.4 (4.4–8.4)	4.8 (2.8–8.3)	NS
n-3 fatty acids (g)	0.8 (0.5–0.9)	0.4 (0.3–0.7)	0.001
SFA (g)	23.4 (15.0–30.7)	16.8 (7.8–23.3)	0.006
Trans fats (g)	1.2 (0.6–2.2)	0.6 (0.2–1.1)	0.002
Cholesterol (mg)	143 (74–197)	89 (59–133)	NS
Vitamin B_1_ (% of the RDA)	102 (65–127)	81 (74–128)	NS
Vitamin B_2_ (% of the RDA)	115 (72–158)	109 (54–144)	NS
Vitamin Β_6_ (% of the RDA)	112 (75–162)	91 (42–122)	0.025
Vitamin Β_12_ (% of the RDA)	112 (55–181)	54 (20–118)	0.010
Vitamin Β_9_ (% of the RDA)	74 (48–110)	64 (39–99)	ΝS
Vitamin A (% of the RDA)	46 (27–75)	37 (20–54)	NS
Vitamin D (% of the RDA)	9 (4–27)	8 (2–32)	NS
Vitamin E (% of the RDA)	30 (15–49)	52 (29–66)	0.009
Calcium (Ca) (% of the RDA)	47 (28–66)	44.5 (28–74)	NS
Iron (Fe) (% of the RDA)	54 (37–82)	80 (54–124)	0.037
Magnesium (Mg) (% of the RDA)	50 (30–59)	63.5 (41–76)	0.010
Phosphorus (P) (% of the RDA)	52 (34–73)	60 (38–83)	NS
Zinc (Zn) (% of the RDA)	54 (30–66)	71 (43–108)	0.025
Sodium (Na) (% of the RDA)	96 (65–152)	138 (93–201)	0.028

EI—energy intake; MUFA—monounsaturated fatty acids; NS—not significant; PUFA—polyunsaturated fatty acids; RDA—recommended daily allowances; SFA—saturated fat intake. * Data are presented as medians with their respective 1st and 3rd IQR.

**Table 3:** Studies assessing vitamin B_12_ status in children and adolescents on MET therapy.

First Author	Origin	Design	Sample	Results
Anderson [30]	Australia	12-month double-blind placebo-controlled RCT	N = 90 children and adolescents, >50th BMI PC, with T1DM	Vitamin B_12_ concentrations were lower in the MET group compared with placebo but were still within the reported reference range, with no change in tHcy concentrations.
Azcona-Sanjulián [24]	Spain	Prospective cohort (6 months)	N = 21 pediatric patients with obesity, unresponsive to lifestyle treatment, on MET	No change was noted in the vitamin B_12_ concentrations after 6 months of MET use.
Burgert [25]	USA	4-month double-blind RCT	N = 28 adolescents with obesity and IR randomized to MET (*n* = 15, dose: 1500 mg/day) or placebo (*n* = 13); all patients received a daily MV with vitamin B_12_	No difference was noted in vitamin B_12_ concentrations between subjects taking MET and those on placebo.
der Aa [27]	Netherlands	18-month RCT	N = 42 adolescents with obesity and IR randomized to MET (*n* = 23, 2000 mg/day) or placebo (*n* = 19) and physical training twice/week	At the end of treatment, 3 patients (13%) in the MET-receiving arm had vitamin B_12_ deficiency.
Gourgari [31]	USA	Case report	N = 1 adolescent girl with obesity and PCOS on MET (2000 mg/day)	The girl exhibited vitamin B_12_ deficiency. Treatment with oral cyanocobalamin was initiated (1000 μg/day). After 1 month, normal B_12_ concentrations were attained. ONS with vitamin B_12_ was discontinued, and the patient returned after 5 months. Serum B_12_ concentrations had decreased, but remained within normal range.
Lentferink [28]	Netherlands	Open-label extension of an 18-month double-blind RCT	N = 31 adolescents with obesity and IR on MET or placebo for 18 months	Low vitamin B_12_ concentrations were observed in 2 participants (1 on MET during RCT and extension and 1 on MET during RCT and placebo, on the extension study).
Levy-Shraga [26]	Israel	Case–control	N = 49 children/adolescents with BMI >85 PC treated with DSRA allocated to MET and vitamin B_12_ (*n* = 31) and those on nothing (*n* = 18)	No difference was noted in the vitamin B_12_ concentrations between the groups.
Taş [18]	Turkey	Prospective cohort (6 and 12 months)	N = 24 patients with T2DM, MetS, or PCOS with IR and/or IGT, treated with MET	At the 6-month follow-up, no difference was noted in the vitamin B_12_, tHcy, MMA, and holo-TC-II levels, although a 0.6% decline in vitamin B_12_ concentrations was noted. At 12 months (*n* = 11 patients: 6 with T2DM and 5 with MetS), no difference was noted in vitamin B_12_, tHcy, MMA, and holo-TC-II concentrations, but a 6% decline in vitamin B_12_, a 5.4% decrease in holo-TC-II, and a 10.9% increase in tHcy concentrations were noted.
Yanovski [29]	USA	6-month RCT with a 6-month follow-up	N = 100 children/adolescents with obesity and IR, randomized to 1000 mg MET (*n* = 53) or placebo (*n* = 47) twice/day	Serum vitamin B_12_ levels remained within the normal range in all subjects throughout the 12-month study, but decreased in the MET-treated arm, compared with the increase observed among placebo-treated children.
Yu [17]	USA	Prospective cohort (6, 12, 24, and 36 months)	N = 151 pediatric patients with >3 months of consecutive MET use	No decrease in vitamin B_12_ concentrations was noted at 6, 12, 24, or 36 months among those treated with MET. A reduction in vitamin B_12_ was only noticeable in patients on a high-MET dose, with good compliance; however, levels remained within the normal range. Of the 151 patients, only 1 demonstrated deficiency after a year of MET use.

BMI—body mass index; DSRA—dopamine and serotonin receptor antagonists; Holo-TC-II—holo-transcobalamin-II; IGT—impaired glucose tolerance; IR—insulin resistance; MET—metformin; MetS—metabolic syndrome; MMA—methylmalonic acid; MV—multivitamin; ONS—oral nutrient supplementation; PC—percentile; PCOS—polycystic ovary syndrome; RCT—randomized controlled trial; T1DM—type 1 diabetes mellitus; T2DM—type 2 diabetes mellitus; tHcy—total homocysteine.

**Table 4:** Characteristics of the participating children and adolescents in each study group *.

Characteristics	MET Arm (*n* = 23)	Controls (*n* = 46)	*p*-Value
Age (years)	15.4 ± 1.6	12.4 ± 2.7	<0.001
Boys/girls (*n*)	5/18	20/26	NS
Body weight (kg)	82.5 ± 18.2	63.0 ± 22.9	0.001
Stature (cm)	165.4 ± 9.8	153.9 ± 13.2	0.001
BMI (kg/m^2^)	30.3 ± 7.8	25.8 ± 6.7	0.032
BMIz	2.16 ± 1.37	1.97 ± 1.52	NS
Caucasian/Roma (*n*)	22/1	45/1	NS
Normal weight/overweight/obese (*n*)	4/7/12	14/9/23	NS
Menstrual disorders/premature adrenarche/thelarche/precocious puberty (*n*)	2/0/0/0	0/4/1/1	
GH deficiency/short stature/microphallus/gynecomasty (*n*)	1/0/0/0	0/1/1/1	
PCOS/prediabetes/IR/hyperglycemia (*n*)	2/3/14/0	1/0/12/1	
Hypothyroidism/thyroid dysfunction (*n*)	0/0	7/1	
Duration of MET use (months)	22.7 ± 13.3	-	
Daily MET dose (mg/day)	1494 ± 443	-	

BMI—body mass index; BMIz—BMI z-score [47]; GH—growth hormone; IR—insulin resistance; MET—metformin; NS—not significant; PCOS—polycystic ovary syndrome. * Data are presented as counts (*n*) or means ± their respective standard deviations.

## Data Availability

All data are available to the corresponding author upon request.
